# Discovery, synthesis and biochemical profiling of purine-2,6-dione derivatives as inhibitors of the human poly(A)-selective ribonuclease Caf1

**DOI:** 10.1016/j.bmcl.2015.07.095

**Published:** 2015-10-01

**Authors:** Gopal P. Jadhav, Ishwinder Kaur, Maryati Maryati, Blessing Airhihen, Peter M. Fischer, G. Sebastiaan Winkler

**Affiliations:** a School of Pharmacy, Centre for Biomolecular Sciences, University of Nottingham, University Park, Nottingham NG7 2RD, UK; b School of Pharmacy, University of Nottingham, East Drive, University Park, Nottingham NG7 2RD, UK

**Keywords:** Ribonuclease, Deadenylase, Caf1/CNOT7, PARN, Mg^2+^ dependent nuclease

## Abstract

Eukaryotic mRNA contains a 3′ poly(A) tail, which plays important roles in the regulation of mRNA stability and translation. Well-characterized enzymes involved in the shortening of the poly(A) tail include the multi-subunit Ccr4-Not deadenylase, which contains the Caf1 (Pop2) and Ccr4 catalytic components, and poly(A)-specific ribonuclease (PARN). Two Mg^2+^ ions present in the active sites of these ribonucleases are required for RNA cleavage. Here, we report the discovery, synthesis and biochemical profiling of purine-2,6-dione derivatives as (sub)micromolar inhibitors of Caf1.

## Graphical abstract

**Figure:**
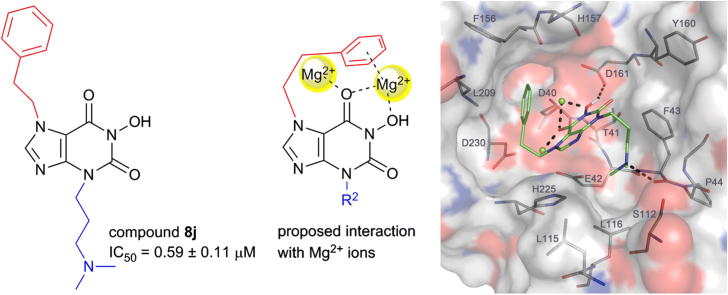

In eukaryotic cells, cytoplasmic mRNA is characterized by the presence of a 3′ poly(A) tail. The median length of the tail varies from 27 to 28 nucleotides in yeast to 60–100 nucleotides in mammalian cells.^1,2^ The tail is important for the control of gene expression: enzymatic shortening of the poly(A) tail (deadenylation) can initiate mRNA degradation and repress translation.^3^ An important enzyme involved in cytoplasmic deadenylation is the multi-component Ccr4-Not complex.^4,5^ In addition to six non-catalytic subunits, the complex contains two subunits with ribonuclease activity: both Caf1 and Ccr4 display Mg^2+^-dependent 3′–5′ *exo*-ribonuclease activity with a preference for poly(A). However, whereas the enzymatic activity of Caf1 is associated with an RNAse D/DEDD (Asp-Glu-Asp-Asp) domain,^6–8^ the enzymatic activity of Ccr4 is provided by an EEP (endonuclease-exonuclease-phosphatase) domain.^9,10^

The analysis of genetically modified mice has identified the importance of Ccr4-Not subunits in the regulation of physiological functions such as bone formation and male fertility^11,12^ as well as obesity and heart disease.^13,14^ Moreover, mutations in *CNOT3* are frequently identified in acute lymphoblastic leukemia.^15^ Currently, it is unclear whether the ribonuclease activities of the Ccr4-Not complex are involved in the regulation of these processes and to what extent the Caf1 and Ccr4 subunits have unique roles, or cooperate in deadenylation.^16–18^ Because the structural complexity of the Ccr4-Not deadenylase is a significant barrier to distinguishing between catalytic and structural roles of the complex, additional tools to study Ccr4-Not function are required. In particular, cell-permeable small-molecule inhibitors that selectively inhibit the enzyme activities of Caf1 or Ccr4 are desirable as a complementary approach to RNAi and genetic techniques. Such molecules will be highly useful as pharmacological tools to study the involvement of the catalytic activity of Ccr4-Not in physiological processes, and contribute to the evaluation of this complex as a potential therapeutic target.

While nucleoside analogues have been reported as inhibitors of the poly(A)-specific ribonuclease PARN, whose DEDD-type nuclease domain is closely related to that of Caf1,^19,20^ non-nucleoside inhibitors have only been reported for a limited number of Mg^2+^-dependent ribonucleases. These include influenza RNA endonuclease (required for viral transcription) and human immunodeficiency virus (HIV) RNAse H (an RNA endonuclease involved in the degradation of RNA strands of RNA:DNA hybrids).^21,22^ In addition, we recently reported the discovery of non-nucleoside inhibitors of Caf1, which were identified by screening a compound library.^22^ Here, we describe an alternative approach, based on the discovery of *N*-hydroxyimide compounds as inhibitors of HIV RNAse H and human flap endonuclease FEN1, a structure-specific DNA endonuclease.^23,24^ It was suggested that these compounds inhibited enzyme activity by coordination of the two divalent metal ions required for catalysis (Fig. 1A). Interestingly, *N*-hydroxyimides were also identified as inhibitors of the influenza RNA endonuclease PA.^21^ Based on the distance between the Mg^2+^ ions in the active site of the Caf1 ribonuclease (3.9–4.0 Å; Fig. 2a and b),^25^ we hypothesized that compounds containing this moiety may adopt a similar binding mode in the active site of Caf1, thereby blocking substrate binding.

We therefore searched the Open Chemical Repository Collection, a diverse set of more than 200,000 compounds (Developmental Therapeutics Program, National Cancer Institute, Bethesda), for the presence of *N*-hydroxyimides. To limit the number of compounds, we focused on 3-hydroxypyrimidine-2,4-diones and obtained seven compounds for further analysis. Following LC–MS and solubility analysis, five compounds were evaluated for their ability to inhibit Caf1 activity using a fluorescence-based biochemical assay (Fig. 1B).^22^ Interestingly, whereas compound NSC-86353 was found to inhibit Caf1 (IC_50_ = 22.8 ± 0.1 μM), substitution of the benzyl moiety with a methyl group at the *N*^7^ position (NSC-85703) reduced the inhibitory activity >10-fold.

Based on this observation, we first explored the activity of 7-substituted 1-hydroxy-purine-2,6-diones **5** further. The synthesis of these compounds was accomplished by amination (with amines **1**) of ethyl 2-bromoacetate to provide the *N*-substituted ethyl 2-aminoacetate intermediates **2** (Scheme 1). These were *N*-alkylated with (ethoxymethylene)cyanamide^26^ and the resulting cyanoformamidine adducts were cyclised in situ^27^ with KOBu*^t^* to the 3-subsituted ethyl 5-aminoimidazole-4-carboxylates **3**. The 1-allyloxy-purinedione system of the bicyclic compounds **4** was then formed by cyclisation of intermediates **3** with the aid of carbonyldiimidazole (CDI) and *O*-allylhydroxylamine.^28^ The target compounds **5** were obtained following reductive deprotection of precursors **4**.^29^

Starting with 7-benzylpurinedione **5a** (Table 1), we hypothesised that the *N*^7^-benzyl substituent binds into the hydrophobic Caf1 active site pocket delineated by residues Phe^156^, Leu^209^, and His^225^ (Fig. 2c and d).^31–33^ Based on this hypothesis, we introduced comparatively bulky lipophilic groups at *N*^7^. Replacement of the benzyl with a cyclohexyl group (**5b**) reduced activity nearly 3-fold, in line with what we observed upon substituting the benzyl with a simple methyl group (see above). More conservative replacements of the benzyl with pyridylmethyl groups afforded active compounds, especially for the 2- and 3-pyridylmethyl analogues **5c** and **5d**, whereas the 4-pyridylmethyl isomer **5e** was less active. Lengthening of the alkyl linker between the phenyl group and *N*^7^ from CH_2_ (**5a**) to (CH_2_)_2_ (**5j**) and (CH_2_)_3_ (**5f**) showed maximal activity for the phenethyl derivative **5j**. This observation is in line with the modelled Caf1 binding poses of compounds containing the phenethyl group (Fig. 2c). These poses suggest an optimal cation–π interaction between one of the Mg^2+^ ions and the phenyl ring in the case of a 2-carbon linker, while maintaining hydrophobic interactions of the phenyl ring with Leu^209^. Similar dual interactions with the Caf1 active site are not possible with 1- and 3-carbon linkers between the phenyl ring and *N*^7^. Replacement of the benzyl group with a long-chain lipophilic substituent (**5g**) was not productive. On the other hand, insertion of an O atom into the linker (**5i**), or replacement of the phenyl group in **5j** with a 2-thienyl (**5h**) or 2-pyridyl (**5k**) group, retained most of the activity.

We next turned our attention to the purine *N*^3^-substituent in the context of the optimal 7-phenethyl group (Table 2). Here we envisaged that a comparatively bulky substituent may make favourable contacts with the Caf1 active site pocket lined by residues Phe^43^, Pro^44^, Ser^112^, and Leu^116^ (Fig. 2c and d). The 3,7-disubstituted 1-hydroxypurine-2,6-diones **8** were prepared from the *O*-benzyl precursor **6**, obtained by cyclisation of imidazole **3** (R^1^ = (CH_2_)_2_Ph) with *O*-benzylhydroxylamine,^34^ by successive alkylation with appropriate alkyl halides and hydrogenolysis of products **7** (Scheme 1). Introduction of a methyl (**8a**), ethyl (**8c**), *n*-pentyl (**8d**), isohexyl (**8e**), *n*-nonyl (**8f**), *n*-decyl (**8g**), or *n*-dodecyl group (**8h**) at *N*^3^ showed optimal activity for the isohexyl congener **8e**, although its activity was not improved with respect to the parent compound **5j**. However, replacement of the isohexyl group with the 1-(*N*,*N*-dimethylamino)prop-3-yl group (**8j**) resulted in a further affinity enhancement and afforded the most potent compound in our series with activity at the submicromolar level.^35^ Again this potency gain can be rationalised based on docking studies, which suggest that the protonated (at physiological pH) 3° amine group in **8j** is H-bonded to the amide carbonyl O of Pro^44^, while maintaining most of the hydrophobic interactions of the *N*^3^-substituent compared with the isohexyl group in **8e** (Fig. 2d).

According to our binding hypothesis (Fig. 2c) the *N*-hydroxyimide function, especially the *C*^6^- and *N*-linked O atoms, present in all compound of the series discussed thus far, is important for polar interactions with the Mg^2+^ ions coordinated in the active site of Caf1. Additionally, we propose that the *N*-hydroxyl function H-bonds to the side-chain carboxyl of Asp^61^. To probe this hypothesis we tested compounds containing modified *N*-hydroxyimide substructures (Table 3). To this end, a purine-2,6-dione derivative (**9**) lacking the *N*-hydroxy group of compounds **5** and **8** was obtained from intermediate **3** (R^1^ = Bn) by cyclisation with urea under basic conditions (Scheme 1).^34^ 6-Hydroxy-1-phenethyl-1,4-dihydro-5*H*-imidazo[4,5-*b*]pyridin-5-one **13** was obtained from the nitroimidazole precursor **10** (Scheme 2).^36^ A dichloromethyl group was introduced into this compound at the 5-position using a vicarious nucleophilic procedure with chloroform and KOBu*^t^*, followed by hydrolysis of the dichloromethyl group to the aldehyde and reduction of the nitro function to the aminoaldehyde **11**.^37^ This intermediate was then cyclised with ethyl allyloxyacetate to the imadazopyridinone **12**,^38^ which was deprotected to afford target compound **13**. Blocking of the *N*-hydroxyl function with an allyl group in the synthesis intermediate **4a**, as well as removing the *N-*hydroxyl group in **9**, abolished activity completely, as expected. Furthermore, omission of the *C*^6^-carbonyl oxygen in the hydroxy-imidazopyridinone **13** reduced activity significantly, pointing to an important contribution of this carbonyl group to binding, as suggested by our docking studies.

The selectivity of the compounds was determined by comparing their activities against Caf1 and two related deadenylase enzymes: the PARN ribonuclease, which has a conserved nuclease domain similar to that of Caf1, and Ccr4, whose biological function is related to Caf1, but which contains a dissimilar EEP-type nuclease domain.^22^ Compounds with *N*^7^ substituents containing an aromatic group linked via a single carbon spacer (**5a** and **5c**–**e**) selectively inhibited Caf1 (Fig. 3; Table 1). By contrast, the more potent compounds **5h**, **5j** and **5k** with *N*^7^ substituents containing an aromatic group linked via a two-carbon spacer facilitating optimal cation–π interactions displayed reduced selectivity and also inhibited the related deadenylase PARN. Selectivity was not improved by the introduction of *N*^3^ allyl substituents (**8a**–**8f**). However, the most potent compound **8j** containing an *N*^3^-1-(*N*,*N*-dimethylamino)prop-3-yl group displayed reduced activity versus PARN, suggesting that substituents in this position can contribute to selectivity (Table 2). None of the compounds tested displayed activity towards the EEP-type deadenylase Ccr4.

The molecules described here are structurally unrelated to the inhibitors of Caf1 that we described before and which were identified using a combined approach involving virtual and compound library screening.^22^ Compound **8j** is significantly more potent (>20-fold) than all previously identified compounds, although the potency of the lead compound NSC-86353 (**5a**) is comparable to that of NCC-00037292 (IC_50_ = 14.6 ± 3.1 μM), which was reported previously.^22^ To the best of our knowledge, no other inhibitors of the Caf1 deadenylase have been described to date.

In summary, we discovered purine-2,6-dione derivatives as inhibitors of the ribonucleases Caf1 and PARN. Compound **8j** was the most potent compound with a >10-fold increased activity as compared to compound **5a**, which was identified as the initial hit compound. In addition to polar interactions involving the *N*-hydroxyimide moiety, we propose that a cation–π interaction contributes to coordination of the Mg^2+^ ions. We conclude that these compounds will be useful for the biochemical analysis of deadenylase enzymes and contribute to the design of improved inhibitors of the Caf1 and PARN ribonucleases with increased potency and selectivity.

## Figures and Tables

**Figure 1:**
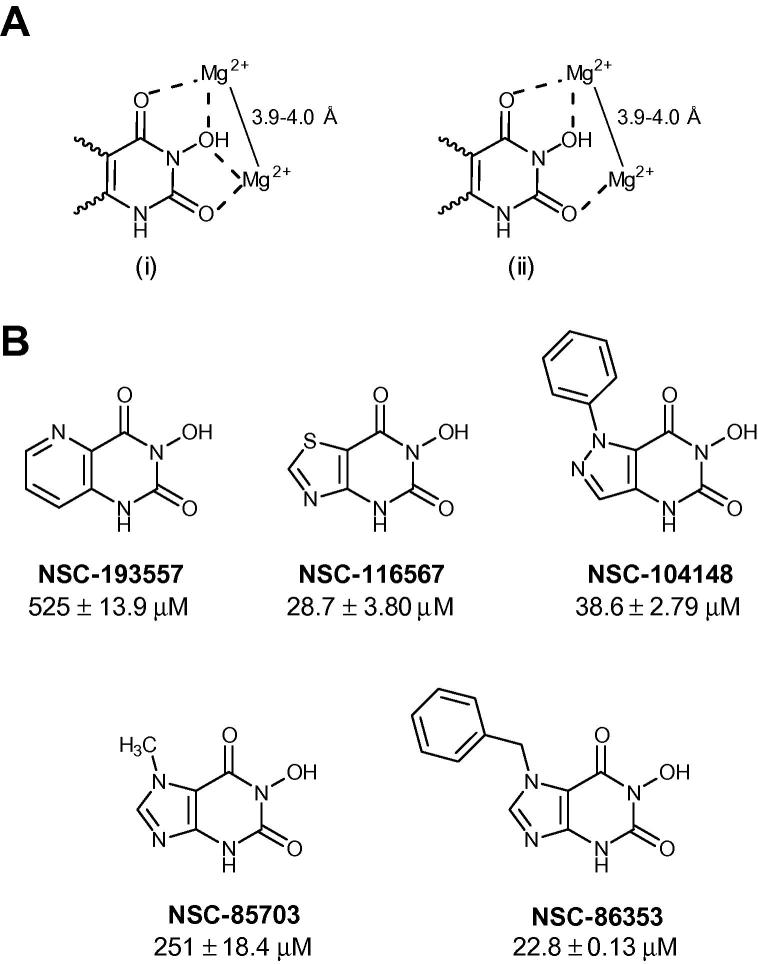
Discovery of 3-hydroxy-pyrimidine-2,4-dione compounds as inhibitors of the Caf1 ribonuclease. (A) Possible interaction modes of 3-hydroxy-pyrimidine-2,4-dione compounds with two divalent metal ions in the active site of Caf1. (B) Structure and activity of compounds selected from the Open Chemical Repository Collection (NCI, Bethesda). IC_50_ values refer to inhibition of Caf1. Also indicated is the standard error of the mean (*n* = 3).

**Figure 2:**
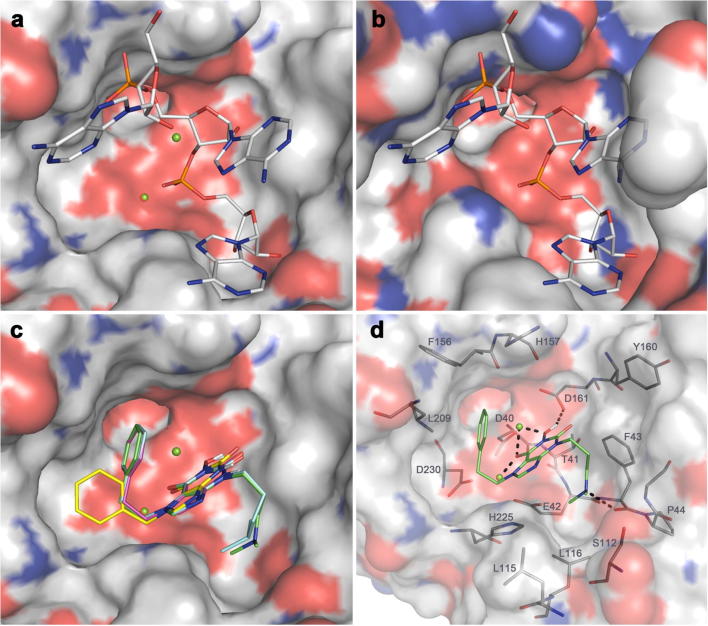
Molecular modelling of inhibitors in the active site of Caf1. Complex crystal structures of human Caf1 (**a**; PDB 4GMJ.B)^25^ and PARN (**b**; PDB 2A1R)^30^ show that the cognate poly(A) ligand (stick models) likely binds in a similar manner to the active site (protein surface with Mg^2+^ ions as green spheres), despite the fact that the active sites in Caf1 and PARN have somewhat different shapes. In (**c**) it can be seen that the top-ranking poses of the most potent compounds **5j** (magenta), **8e** (cyan), and **8j** (green) adopt very similar conformations upon docking to the 4GMJ.B model, whereas several poses with similar scores were recorded for lead compound **5a** (yellow), whereof only some (one shown) were similar to the bound conformations of the more potent compounds. Probable polar binding contacts of the most potent compound **8j** with the Caf1 active site include a cation–π interaction between one of the Mg^2+^ ions and the phenyl ring of the inhibitor, as well as H-bonds and polar interactions involving the 3° amine and one of the carbonyl O and the OH of the *N*-hydroxyimide group as indicated (**d**; dashed lines). Furthermore, **8j** makes numerous van-der-Waals contacts with residues (labelled in **d**) of the active site cavity.

**Figure 3:**
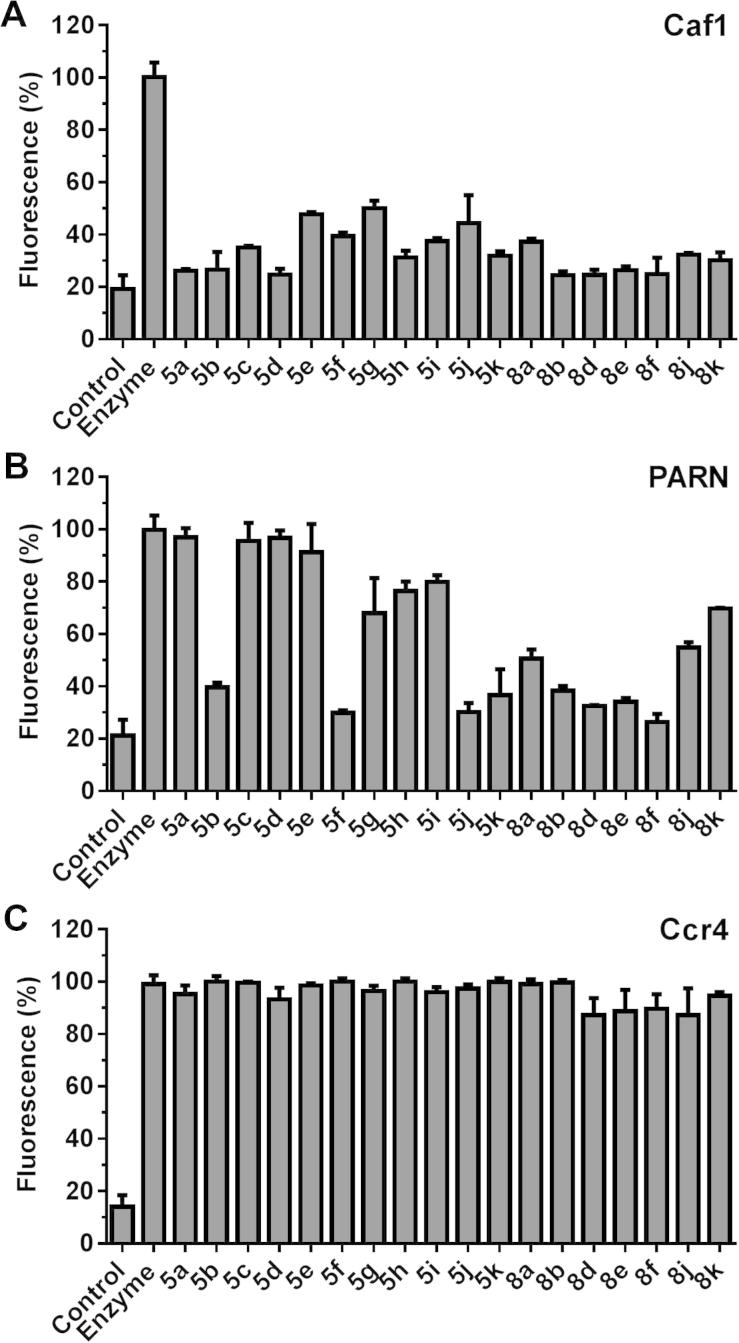
Inhibition of the ribonuclease activities of (A) Caf1, (B) PARN and (C) Ccr4 by 3,7-*N*-substituted-1-hydroxy-3,7-dihydro-1*H*-purine-2,6-diones. Reactions contained no enzyme (control), complete reactions (enzyme) or complete reactions in the presence of the indicated compound. Compounds were used at 30 μM (**5a**–**e**, **8a**–**d**, **8f**), 10 μM (**5h**–**k**), or 3 μM (**8e**, **8j**). Error bars represent the standard deviation.

**Scheme 1:**
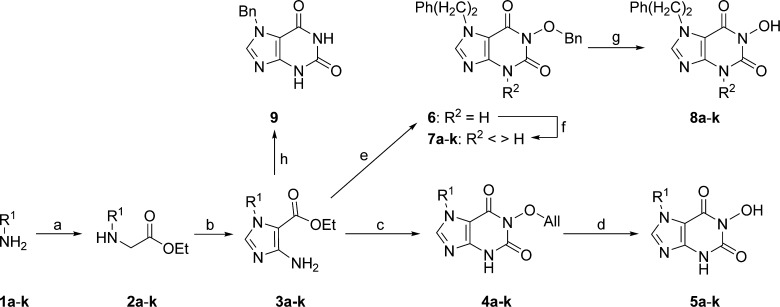
Synthesis of 7-substituted 1-hydroxy-3,7-dihydro-1*H*-purine-2,6-diones. Reagents and conditions: (a) ethyl 2-bromoacetate, CHCl_3_, rt, 2 h (37–79%); (b) (ethoxymethylene)cyanamide, THF, Δ, 16 h, then KOBu*^t^*, EtOH, Δ, 2 h (46–80% over 2 steps); (c) CDI, PhMe, Δ, 2 h, then *O*-allylhydroxylamine, aq NaOH, EtOH, Δ, 2 h (21–61% over 2 steps); (d) Pd(OAc)_2_, PPh_3_, HCOOH, EtOH–H_2_O (8:2), 80 °C, 2 h (23–55%); (e) CDI, PhMe, Δ, 2 h, then *O*-benzylhydroxylamine, aq NaOH, EtOH, Δ, 2 h (74% over 2 steps); (f) R^2^X (X = Br or I), K_2_CO_3_, DMF, 80 °C, 2–12 h, (67–98%); (g) H_2_, 10% (w/w) Pd(C), CH_2_Cl_2_–MeOH (1:9) (33–53%); (h) urea, 2-methoxyethanol, 190 °C, 24 h, then aq NaOH, Δ, 3 h (33%). For definitions of R^1^ and R^2^ refer to Tables 1–3.

**Scheme 2:**
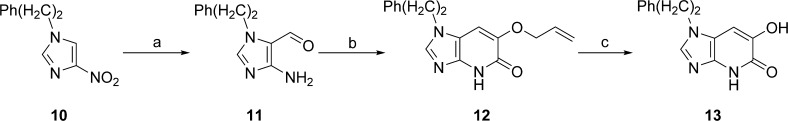
Synthesis of 6-hydroxy-1-phenethyl-1,4-dihydro-5*H*-imidazo[4,5-*b*]pyridin-5-one **13**. Reagents and conditions: (a) CHCl_3_, KOBu*^t^*, THF–DMF (2:1), −100 °C, 15 min, then CaCO_3_, H_2_O, 75 °C, 48 h, then H_2_, 10% (w/w) Pd(C), EtOAc–MeOH–AcOH (18:1:1), 55 psi, 18 h (60% over 3 steps); (b) ethyl allyloxyacetate, (Me_3_Si)_2_NLi, THF, −78 °C to rt over 16 h (36%); (c) Pd(OAc)_2_, PPh_3_, HCOOH, EtOH–H_2_O (8:2), 80 °C, 2 h (33%).

**Table 1:** Activity of 7-substituted 1-hydroxy-3,7-dihydro-1*H*-purine-2,6-diones **5**

Cmpd		IC_50_ (μM)^a^ (Caf1)	IC_50_ (μM)^a^ (PARN)
**5a**		10.4 ± 0.4	84.1 ± 6.7
**5b**		28.2 ± 7.6	n.d.
**5c**		10.6 ± 2.7	119 ± 25
**5d**		6.6 ± 0.7	125 ± 32
**5e**		23.3 ± 2.2	245 ± 20
**5f**		13.3 ± 2.3	n.d.
**5g**		20.6 ± 5.8	n.d.
**5h**		3.6 ± 1.1	n.d.
**5i**		10.6 ± 4.3	n.d.
**5j**		1.5 ± 0.3	n.d.
**5k**		4.0 ± 1.1	n.d.

a IC_50_ values were determined using a fluorescence-based biochemical assay as described.^22^ Also indicated are the standard errors of the means (*n* = 3). N.d., not determined.

**Table 2:** Activity of 3-substituted-1-hydroxy-7-phenethyl-3,7-dihydro-1*H*-purine-2,6-diones **8**

Cmpd		IC_50_^a^ (μM) (Caf1)	IC_50_^a^ (μM) (PARN)
**8a**	CH_3_	9.3 ± 1.7	n.d.
**8b**	CH_2_(CH_2_)_2_Ph	2.1 ± 0.3	n.d.
**8c**	CH_2_CH_3_	12.2 ± 3.5	n.d.
**8d**	CH_2_(CH_2_)_3_CH_3_	4.8 ± 1.2	n.d.
**8e**		1.7 ± 0.4	n.d.
**8f**	CH_2_(CH_2_)_7_CH_3_	9.9 ± 3.6	n.d.
**8g**	CH_2_(CH_2_)_8_CH_3_	20.7 ± 6.7	n.d.
**8h**	CH_2_(CH_2_)_10_CH_3_	29.5 ± 22.4	n.d.
**8i**	CH_2_Ph	14.5 ± 0.9	n.d.
**8j**		0.59 ± 0.11	23.9 ± 3.7
**8k**		8.7 ± 0.3	n.d.

a IC_50_ values were determined using a fluorescence-based biochemical assay as described.^22^ Also indicated are the standard errors of the means (*n* = 3). N.d., not determined.

**Table 3:** Activity of 7-substituted 1-hydroxy-3,7-dihydro-1*H*-purine-2,6-diones with modified *N*-hydroxyimide function

Cmpd	Structure	IC_50_^a^ (μM) (Caf1)
**4a**		>1000
**9**		>1000
**13**		15.1 ± 0.3

a IC_50_ values were determined using a fluorescence-based biochemical assay as described.^22^ Also indicated are the standard errors of the means (*n* = 3).

## Supplementary data

The following ZIP file contains the MOL files of the most important compounds referred to in this article.

MOL files ZIP file containing the MOL files of the most important compounds in this article.
